# Life Course Trajectories of Systolic Blood Pressure Using Longitudinal Data from Eight UK Cohorts

**DOI:** 10.1371/journal.pmed.1000440

**Published:** 2011-06-14

**Authors:** Andrew K. Wills, Debbie A. Lawlor, Fiona E. Matthews, Avan Aihie Sayer, Eleni Bakra, Yoav Ben-Shlomo, Michaela Benzeval, Eric Brunner, Rachel Cooper, Mika Kivimaki, Diana Kuh, Graciela Muniz-Terrera, Rebecca Hardy

**Affiliations:** 1 Medical Research Council Unit for Lifelong Health and Ageing, University College London, London, United Kingdom; 2 Medical Research Council Centre for Causal Analyses in Translational Epidemiology, School of Social and Community Medicine, University of Bristol, Bristol, United Kingdom; 3 Medical Research Council Biostatistics Unit, University of Cambridge, Cambridge, United Kingdom; 4 Medical Research Council Epidemiology Resource Centre, University of Southampton, Southampton, United Kingdom; 5 School of Social and Community Medicine, University of Bristol, Bristol, United Kingdom; 6 Medical Research Council/Chief Scientist Office Social and Public Health Sciences Unit, University of Glasgow, Glasgow, United Kingdom; 7 Department of Epidemiology and Public Health, University College London, London, United Kingdom; Bart's and The London School of Medicine and Dentistry, United Kingdom

## Abstract

**Background:**

Much of our understanding of the age-related progression of systolic blood pressure (SBP) comes from cross-sectional data, which do not directly capture within-individual change. We estimated life course trajectories of SBP using longitudinal data from seven population-based cohorts and one predominantly white collar occupational cohort, each from the United Kingdom and with data covering different but overlapping age periods.

**Methods and Findings:**

Data are from 30,372 individuals and comprise 102,583 SBP observations spanning from age 7 to 80+y. Multilevel models were fitted to each cohort. Four life course phases were evident in both sexes: a rapid increase in SBP coinciding with peak adolescent growth, a more gentle increase in early adulthood, a midlife acceleration beginning in the fourth decade, and a period of deceleration in late adulthood where increases in SBP slowed and SBP eventually declined. These phases were still present, although at lower levels, after adjusting for increases in body mass index though adulthood. The deceleration and decline in old age was less evident after excluding individuals who had taken antihypertensive medication. Compared to the population-based cohorts, the occupational cohort had a lower mean SBP, a shallower annual increase in midlife, and a later midlife acceleration. The maximum sex difference was found at age 26 (+8.2 mm Hg higher in men, 95% CI: 6.7, 9.8); women then experienced steeper rises and caught up by the seventh decade.

**Conclusions:**

Our investigation shows a general pattern of SBP progression from childhood in the UK, and suggests possible differences in this pattern during adulthood between a general population and an occupational population.

*Please see later in the article for the Editors' Summary*

## Abstract 2

Analysis of eight population-based and occupational cohorts from the UK reveals the patterns of change of blood pressure in the population through the life course.

## Editors' Summary

**Background:**

About a third of US and UK adults have high blood pressure (hypertension). Although hypertension has no obvious symptoms, it can lead to life-threatening heart attacks, stroke, and other forms of cardiovascular disease (CVD). It is diagnosed by measuring blood pressure—the force that blood moving around the body exerts on the inside of large blood vessels. Blood pressure is highest when the heart is pumping out blood (systolic blood pressure [SBP]) and lowest when the heart is re-filling with blood (diastolic blood pressure [DBP]). Normal adult blood pressure is defined as an SBP of less than 130 millimeters of mercury (mm Hg) and a DBP of less than 85 mm Hg (a blood pressure of 130/85). A reading of more than 140/90 indicates hypertension. Many factors affect blood pressure, but overweight people and individuals who eat fatty or salty food are at high risk of developing hypertension. Moreover, blood pressure tends to increase with age. Mild hypertension can often be corrected by making lifestyle changes, but many people take antihypertensive drugs to reduce their blood pressure.

**Why Was This Study Done?:**

Several trials have indicated that SBP is an important, modifiable risk factor for CVD. But, to determine the best way to prevent CVD, it is important to understand how SBP changes through life and how lifestyle factors affect this age-related progression. Textbook descriptions of age-related changes in SBP are based on studies that measured SBP at a single time point in groups (cohorts) of people of different ages. However, such “cross-sectional” studies do not capture within-individual changes in SBP and may be affected by environmental effects related to specific historical periods. The best way to measure age-related changes in SBP is through longitudinal studies in which SBP is repeatedly measured over many years in a single cohort. Such studies are underway, but it will be some decades before individuals in these studies reach old age. In this study, therefore, the researchers use data from multiple UK cohorts that had repeated SBP measurements taken over different but overlapping periods of life to investigate the life course trajectory of SBP.

**What Did the Researchers Do and Find?:**

The researchers used statistical models to analyze data from longitudinal studies of SBP in seven population-based cohorts (the participants were randomly chosen from the general population) and in one occupational cohort (civil servants). SBP measurements were available for 30,372 individuals with ages spanning from seven years to more than 80 years. The researchers' analysis revealed four phases of SBP change in both sexes: a rapid increase in SBP during adolescent growth, a gentler increase in early adulthood, a midlife acceleration beginning in the fourth decade of life, and a period in late adulthood when SBP increases slowed and then reversed. This last phase was less marked when people taking antihypertensive drugs were excluded from the analysis. After adjusting for increases in body mass index (a measure of body fat) during adulthood, the magnitude of the SBP age-related changes was similar but the average SBP at each age was lower. Compared to the population-based cohorts, the occupational cohort had a lower average SBP, a shallower annual increase in SBP, and a later midlife acceleration, possibly because of socially determined modifiable SBP-related factors such as diet and lifestyle. Finally, although women had lower SBPs in early adulthood than men, they experienced steeper midlife SBP rises (probably because of a menopause-related effect on salt sensitivity) so that by the seventh decade of life, men and women had similar average SBPs.

**What Do These Findings Mean?:**

These findings describe the general pattern of age-related progression of SBP from early childhood in the UK. The findings may not be generalizable because other populations may be exposed to different distributions of modifiable factors. In addition, their accuracy may be affected by differences between cohorts in how SBP was measured. Nevertheless, these findings—in particular, the slower midlife increase in SBP in the occupational cohort than in the population-based cohorts—suggest that the key determinants of age-related increases in blood pressure are modifiable and could be targeted for CVD prevention. Further research is now needed to identify exactly which factors affect the life course trajectory of SBP and to discover when these factors have their greatest influence on SBP.

**Additional Information:**

Please access these Web sites via the online version of this summary at http://dx.doi.org/10.1371/journal.pmed.1000440.

- The US National Heart Lung and Blood Institute has patient information about high blood pressure (in English and Spanish)
- The American Heart Association provides information on high blood pressure and on cardiovascular diseases (in several languages)
- The UK National Health Service Choices Web site also provides detailed information for patients about hypertension and about cardiovascular disease
- MedlinePlus provides links to further information about high blood pressure, heart disease, and stroke (in English and Spanish)

## Introduction

Systolic blood pressure (SBP) is an important indicator of cardiovascular function as it has a strong, positive, and continuous relationship with cardiovascular disease (CVD) and mortality [1]. In prospective studies that measured SBP in adolescence or early adulthood, SBP has been shown to predict future risk of CVD with the same magnitude of association as that seen in studies measuring blood pressure (BP) in middle age [2]–[5]. Despite a tendency for SBP to track through life [6],[7], randomised controlled trials demonstrate that it is a highly modifiable risk factor [8]. Understanding the progression of SBP through life and factors affecting this progression is clearly important to determining the best methods for preventing future CVD.

Textbook descriptions of age-related changes in BP are based on cross-sectional studies of different age groups [9]. These show marked increases in SBP in the first year of life (from ∼70 mm Hg to 95 mm Hg), followed by steady increases of 1–2 mm Hg per year to the late 20 s, after which the rise is more marked to age 80, when it begins to level off. Cross-sectional data cannot directly measure within-individual change, and thus the tempo of age-related BP may be misrepresented by the monotonic secular decline in BP over the last six decades [10]–[12], also called “cohort” effects, or by contextual changes related to specific historical periods. Whilst there are a small number of published population-based longitudinal studies on age-related BP changes in children [13] and adults [14]–[16], to date no study to our knowledge has repeat BP measurements from childhood to late adulthood, and it will be some decades before existing birth cohorts that do have repeat infant and childhood measurements reach middle and old age. An alternative to examining life course trajectories in a single cohort, and a potential improvement over cross-sectional analyses, is to compare data from multiple cohorts with repeated measurements that cover different and overlapping periods of life.

We obtained longitudinal data on BP from eight UK Medical Research Council funded cohort studies, each covering different age periods. The objectives of this paper are to (1) describe the average unadjusted SBP trajectory in each cohort, (2) examine the potential to which these trajectories are modifiable by adjusting for a strong determinant of SBP, adiposity, as marked by concurrent body mass index (BMI) [8],[17], and (3) investigate gender differences in SBP trajectories. In addressing these questions, we also explore the methodological issues that arise when using multiple cohorts to investigate life course BP.

## Methods

### Study Population

All cohorts included in these analyses were receiving funds from the UK Medical Research Council and had at least two repeat measurements of BP. Seven cohorts drawn from the general population, in the sense that they were sampled in a way that made them approximately representative of a geographical area, were included. These were the Caerphilly Prospective Study (CaPS), which includes only men [18], the Hertfordshire Ageing Study (HAS) [19], the Medical Research Council National Survey of Health and Development (NSHD) (1946 British birth cohort) [20], three cohorts from the West of Scotland Twenty-07 study (T-07) [21], and the Avon Longitudinal Study of Parents and Children (ALSPAC) [22]. The Whitehall II study (WHII) [23], which is an occupational cohort, was the eighth study included. All cohorts received ethical approval [18]–[23]. The NSHD is in most respects a nationally representative sample from England, Scotland, and Wales [20] while the other cohorts were sampled from single towns, cities, or counties in the UK. The T-07 study comprises three separate cohorts born 20 y apart (1932/1933, 1952/1953, 1972/1973) with measures across the three cohorts occurring at similar dates. Two sampling schemes were used in the T-07 study; for these analyses we used data from the regional sample [21] as it is population representative and was followed up at all waves of data collection. WHII is a prospective cohort study of civil servants aged 35 to 55 y working in the London-based offices of 20 Whitehall departments [23].

### Blood Pressure Measurement

Table 1 describes the BP measurement protocols in each cohort. All studies used trained nurses or fieldworkers, a seated posture, appropriate cuff sizes for arm circumference, and allowed at least 2 min rest prior to measurement. ALSPAC and HAS used an automated oscillometric (AO) device. CaPS, NSHD, T-07, and WHII used a manual random zero sphygmomanometer (MRZ) at earlier waves and switched to an AO device for the most recent waves of data collection (Table 1). To make the BP readings from separate waves within each cohort comparable, we used published equations [24] to convert the AO measurements at wave 5 in CaPS, wave 3 in NSHD, waves 4 and 5 in T-07, and wave 4 in WHII to an MRZ value. Failing to correct for a device switch leads to a biased trajectory by making the trajectory steeper between ages where the device was switched (see Text S1). However, while SBP tends to be higher when measured with an AO compared to MRZ device, a sensitivity analysis using the T-07 cohorts, which have three repeat measures with each type of device, suggested that the slopes of the trajectories were largely unaffected by device type (see Text S1). At least two readings were taken at each wave in all cohorts except CaPS. In the T-07 cohorts at wave 3, the first reading was taken with a MRZ and the second with an AO device. To maintain consistency between cohorts, we report results from the first reading. A sensitivity analysis using the second reading where available showed that this choice did not qualitatively alter our findings on the overall life course trajectory (see Text S1).

**Table 1:** Blood pressure measurement protocols used in each cohort at each wave.

Protocol Feature	Study					
	ALSPAC	T-07	NSHD	CaPS	HAS	WHII
Posture	Seated	Seated	Seated	Seated	Seated	Seated
Operator	Trained field workers	Nurse	Nurse	Waves 1 to 4: physician; wave 5: trained field worker	Nurse	Nurse
Minimum rest before reading	2 min	5 min	5 min	5 min	5 min	5 min
Number of readings	Waves 1 to 6: 2	Wave 1: 2	Waves 1 to 3: 2	Wave 1: 1	Wave 1: 2	Waves 1 to 4: 2
		Wave 2: 2		Wave 2: 2	Wave 2: 3	
		Wave 3: 2		Wave 3: 1		
		Wave 4: 2		Wave 4: 1		
		Wave 5: 3		Wave 5: 1		
BP device^a^	Waves 1 to 6: AO(D)	Wave 1: MRZ	Wave 1: MRZ	Wave 1: MRZ	Wave 1: AO(D)	Wave 1: MRZ
		Wave 2: MRZ	Wave 2: MRZ	Wave 2: MRZ; A(C)	Wave 2: AO(D)	Wave 2: MRZ
		Wave 3: MRZ; AO(O)	Wave 3: AO(O)	Wave 3: MRZ		Wave 3: MRZ
		Wave 4: AO(O)		Wave 4: MRZ		Wave 4: AO(O)
		Wave 5: AO(O)		Wave 5: AO(O)		

a MRZ, Hawksley MRZ (auscultatory); AO(D), AO (Dinamap); AO(O), AO (Omron); AO(C), AO (Copal UA-231).

### Blood Pressure Medication

Nurses or trained field workers recorded any prescribed medications, and antihypertensive drugs (HypRx) were subsequently coded using the British National Formulary books. In ALSPAC, none of the children had taken antihypertensive medication. Medication data were unavailable for wave 2 in the youngest T-07 cohort (1972/1973) (mean age: 18.6 y), so we made the assumption that none were taking medication at this age given that none of this cohort were on HypRx at the first wave and fewer than 1.2% were on HypRx at wave 3.

### Cohort Characteristics

At each wave, height and weight were measured and BMI was calculated (weight [in kilograms]/height [in meters]^2^). Adult socioeconomic position (SEP) was defined based on own occupation for men and on the husband's occupation for women or woman's own occupation where no data existed for the husband or the woman was unmarried. SEP was defined according to the Registrar General's classification system.

### Analysis

#### Unadjusted life course pattern of SBP (aim 1)

To describe the distribution of SBP across age and between cohorts, the observed median and 10^th^ and 90^th^ centiles were calculated at each wave. To estimate the mean SBP trajectory as a function of age, multilevel models were fitted to each cohort. Cubic and quadratic polynomials were used to describe non-linear trajectories (more details on the modelling strategy are in Text S2). To account for the influence of HypRx on the SBP trajectory, a constant of 10 mm Hg was added to SBP values that were observed while on treatment. This value was selected on the basis of previously reported estimates for the effect of medication on reducing BP [25]–[27], though our findings were robust to the choice of constant (see Text S1). The approach assumes that treatment effects are the same across age, period, and cohort, but has been shown to be a reasonable way of reducing treatment bias [25] and was adopted because we were interested in the age-related progression of SBP in the general population unaffected by the increasing prevalence of HypRx use. Likewise, excluding or censoring individuals on treatment would omit an important subgroup from the population we wish to describe. A sensitivity analysis also showed that our findings in the unadjusted models were unlikely to be biased under an assumption that the data are missing at random (see Text S3).

#### Modifiability of trajectories (aim 2)

As BMI generally increases with age, to see how these increases might influence the age-related SBP trajectory, models were fitted including BMI as a time-varying covariate, adjusting the SBP trajectory as if BMI remained at 23 kg/m^2^ through adult life. This value was chosen because it approximates the median BMI at the final age of the UK 1990 growth reference (age 23 y) [28] and is within recommendations for normal weight. For the cohorts where data collection began in childhood (ALSPAC and T-07 1972/1973), we adjusted the SBP trajectory to the median age- and sex-specific BMI values from the UK 1990 growth reference [28]. We also adjusted for baseline height differences in these cohorts using the same growth reference. Text S2 contains more details on these models.

#### Gender differences (aim 3)

Differences between the sexes were tested and described by fitting models that included sex interactions on all the fixed effects of each model; this was done for both the unadjusted and adjusted models.

A restricted maximum likelihood algorithm was used for estimation, and Stata (version 10) was used for all analyses.

## Results

Data are from 30,372 individuals comprising 102,583 data points (Table 2). Each cohort was overlapped to some extent by at least one other cohort with data at a similar age. The birth dates of the cohorts spanned the years 1918 to 1992, and BP data were collected over a 29-y period from 1979 to 2008 (Table 3). The oldest T-07 cohort (1932/1933) and the HAS and CaPS cohorts had the highest proportion of individuals working in manual occupations (Table 3) and were more likely to be from the manual social classes in childhood, reflecting secular changes in the UK labour market. WHII is predominantly a white collar cohort, with less than 10% employed in manual occupations and none in classes IV and V.

**Table 2:** Number of participants and median age (years) at each wave in each population-based cohorts, stratified by sex.

Sex	Study	Childhood							Early Adulthood			Mid Adulthood				Late Adulthood		Totals	
**Male**	ALSPAC	Age	7.5	9.8	10.6	11.7	12.8	15.3											
		*n*	4,139	3,762	3,666	3,455	3,299	2,426											20,747
	T-07 1972/1973	Age						15.7	18.6	24	29.6	36.6							
		*n*						459	431	313	266	286							1,755
	T-07 1952/1953	Age										36	40	44.5	49.7	57			
		*n*										409	373	322	305	312			1,721
	NSHD	Age										36	43	53					
		*n*										1,637	1,598	1,449					4,684
	WHII	Age										43	49	54.8	60.2				
		*n*										6,754	5,522	4,573	4,536				21,385
	T-07 1932/1933	Age												55.9	59.1	63.5	68.8	75.9	
		*n*												443	400	318	241	189	1,591
	CaPS	Age												52.8	57.7	62.4	65.9	73.4	
		*n*												2,507	2,366	2,046	1,793	863	9,575
	HAS	Age															67.5	76.8	
		*n*															454	172	626
																		*n* (male)	62,084
**Female**	ALSPAC	Age	7.5	9.8	10.6	11.8	12.8	15.4											
		*n*	4,024	3,866	3,778	3,576	3,426	2,659											21,329
	T-07 1972/1973	Age						15.7	18.6	24	29.6	36.6							
		*n*						489	475	347	303	335							1,949
	T-07 1952/1953	Age										36.1	40.1	44.5	49.7	56.9			
		*n*										514	475	417	359	360			2,125
	NSHD	Age										36	43	53					
		*n*										1,649	1,590	1,477					4,716
	WHII											44	50	55.6	60.8				
												2,707	2,044	1,620	1,625				7,996
	T-07 1932/1933	Age												56	59.2	63.6	68.8	76	
		*n*												530	456	385	293	234	1,898
	HAS	Age															67.1	76	
		*n*															363	120	483
																		*n* (female)	40,496
																		*n* (all)	102,580

**Table 3:** Cohort information and baseline characteristics by sex.

Characteristic	ALSPAC	T-07 1972/1973	T-07 1952/1953	NSHD	T-07 1932/1933	CaPS	HAS	WHII
*n* males^a^	4,876	478	428	1,841	456	2,951	454	6,892
*n* females^a^	4,815	514	529	1,820	542	—	363	3,413
Year(s) of birth	1991–1992	1972–1973	1952–1953	1946	1932–33	1918–1939	1920–1930	1930–1953
Age range, years^b^	7–16	15–37	34–60	36–53	55–77	44–83	63–81	35–75
Number of waves	7	5	5	3	5	5	2	4
Years of data collection	1998–2008	1987–2008	1987–2008	1982–1999	1987–2008	1979–2005	1994–2005	1985–2004
Population	Children of women attending antenatal clinics in three health districts of Bristol.[22]	Stratified sample from Central Clydeside, Greater Glasgow, Scotland [21]	Stratified sample from Central Clydeside, Greater Glasgow, Scotland [21]	UK representative [20]	Stratified sample from Central Clydeside, Greater Glasgow, Scotland [21]	All men aged 45–59 y living in Caerphilly, Wales [18]	Permanent residents of North Hertfordshire [19]	Civil servants based in 20 offices in Whitehall, London [23]
**Baseline characteristics**								
**Males**								
BMI (kg/m^2^), median (IQR)	0.13 (UK90)^c^	0.07 (UK90)^c^	25.0 (22.8, 27.1)	24.6 (22.7, 26.7)	26.0 (23.6, 28.4)	26.1 (23.9, 28.2)	26.5 (24.1, 29.1)	24.3 (22.6, 26.2)
Height (m), mean (sd)	0.19 (UK90)^c^	−0.09 (UK90)^c^	1.74 (0.07)	1.75 (0.07)	1.71 (0.07)	1.71 (0.06)	1.72 (0.07)	1.76 (0.07)
**SEP, ** ***n*** ** (%)** d								
I	644 (10.8)	40 (8.4)	47 (10.9)	246 (11.5)	23 (4.9)	117 (4.0)	30 (6.6)	2,647 (38.4)
II	1,990 (33.5)	95 (19.8)	122 (28.2)	769 (35.9)	107 (22.6)	482 (16.5)	115 (25.3)	3,607 (52.3)
III—non-manual	732 (12.3)	72 (15.0)	61 (14.1)	213 (9.9)	41 (8.7)	297 (10.2)	40 (8.8)	505 (7.3)
III—manual	1,821 (30.6)	172 (35.9)	141 (32.6)	644 (30.0)	185 (39.1)	1,455 (49.8)	169 (37.1)	136 (2.0)
IV	591 (9.9)	71 (14.8)	41 (9.5)	204 (9.5)	80 (16.9)	422 (14.4)	90 (19.8)	0 (0)
V	170 (2.9)	29 (6.1)	20 (4.6)	68 (3.2)	37 (7.8)	150 (5.1)	11 (2.4)	0 (0)
**Females**								
BMI (kg/m^2^), median (IQR)	0.13 (UK90)^c^	0.12 (UK90)^c^	23.2 (21.3, 26.0)	22.7 (20.9, 25.2)	25.0 (22.6, 28.1)		26.6 (24.0, 29.7)	24.0 (22.5, 28.0)
Height (m), mean (sd)	0.14 (UK90)^c^	−0.2 (UK90)^c^	1.60 (0.06)	1.62 (0.06)	1.59 (0.07)	—	1.59 (0.06)	1.62 (0.07)
**SEP, ** ***n*** ** (%)** d								
I	581 (10.4)	36 (7.0)	39 (7.7)	204 (9.9)	48 (8.5)		29 (8.1)	381 (11.2)
II	1,894 (33.7)	99 (19.3)	125 (24.7)	715 (34.5)	11 (19.5)		74 (20.6)	1,336 (39.1)
III—non-manual	684 (12.2)	68 (13.3)	88 (17.4)	253 (12.2)	97 (17.1)		52 (14.4)	991 (29.0)
III—manual	1,711 (30.5)	196 (38.3)	168 (33.2)	615 (29.7)	170 (29.9)		126 (35.0)	705 (20.7)
IV	583 (10.4)	74 (14.5)	69 (13.6)	213 (10.3)	91 (16.0)		57 (15.8)	0 (0)
V	162 (2.9)	39 (7.6)	17 (3.4)	72 (3.5)	51 (9.0)		22 (6.1)	0 (0)

a The number of participants with at least one BP measurement.

b The 1^st^ centile of wave 1 and 99^th^ centile of the last data collection wave.

c Referenced to the UK 1990 growth reference in *z-*score units [28].

d This is father's SEP for the ALSPAC and T-07 1972/1973 cohorts.

IQR, inter-quartile range; sd, standard deviation.

**Figure 1:**
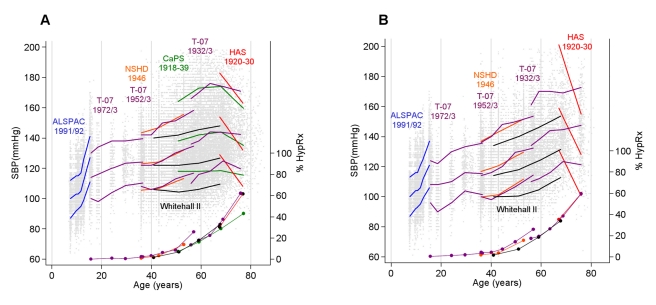
Observed SBP and prevalence of antihypertensive therapy. Observed median and 10^th^ and 90^th^ centiles for SBP (in millimetres of mercury) at each wave in each cohort (for the CaPS and WHII cohorts this is the median in 10-y intervals to allow for the wide age distribution at each wave) and the prevalence (percent) of HypRx use (filled circles) in men (A) and women (B). Individual SBP data points are also plotted. Data presented here do not include an added constant to account for BP medication.

The prevalence of individuals on HypRx was similar in men and women, rising sharply from ∼40 y (Figure 1). For example, in the T-07 1932/1933 cohort, 13% were on medication at 55 y, and 62% at 75 y. For a given age, treatment was more prevalent in the more contemporary cohorts, although the pattern of uptake with age was consistent across cohorts. Figure 1 also shows the observed distribution of SBP with age in each cohort. The distribution widened with age in both sexes from the fourth to seventh decade, as illustrated by comparing waves 1 to 3 between the T-07 1952/1953 and 1932/1933 cohorts where the same measurement device was used.

### Unadjusted SBP Life Course Trajectories

**Figure 2:**
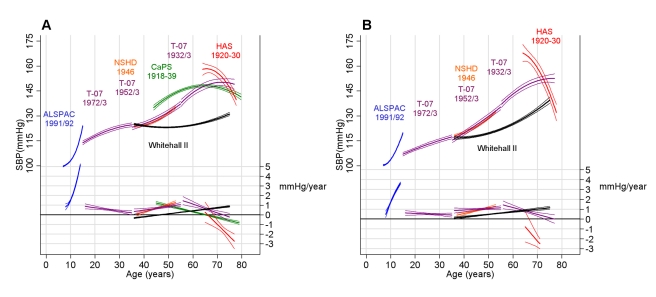
Predicted SBP from unadjusted models. Predicted mean SBP trajectories (in millimetres of mercury) and velocities (millimetres of mercury per year) estimated from unadjusted multilevel models in men (A) and women (B) in each cohort. The thin lines are the 95% CIs.

Figure 2 shows the predicted mean SBP trajectories and annual SBP change estimated from the unadjusted models in each cohort, while Table 4 shows the coefficients for the age effects from these models. The steepest rises in SBP were in adolescence, reaching 5.2 mm Hg per year (95% CI: 5.1, 5.3) in ALSPAC boys and 3.6 mm Hg per year (95% CI: 3.5, 3.8) in ALSPAC girls from 14 to 15 y. From 15 to 30 y, the annual rate of change decreased in T-07 men from 0.9 mm Hg per year (95% CI: 0.6, 1.1) at age 15 y to 0.2 mm Hg per year (95% CI: −0.0, 0.4) at age 35 y. In T-07 women there was a relatively stable linear increase of 0.5 mm Hg per year (95% CI: 0.4, 0.6) from 15 to 35 y. In both men and women, there was then evidence of a midlife SBP acceleration beginning at ∼35 y, and reaching a velocity of ∼1.0 mm Hg per year by age 50 in both the T-07 1952/1953 and NSHD cohorts. The rate of SBP change reached a midlife peak at 55 y of 1.5 mm Hg per year (95% CI: 1.1, 1.9) in men and 1.4 mm Hg per year (95% CI: 1.1, 1.8) in women of the T-07 1932/1933 cohort. SBP increases then slowed and eventually began to decline at age 65, 66, and 70 y in the CaPS, HAS, and T-07 1932/1933 male cohorts, respectively, and at 77 y in the T-07 1932/1933 female cohort. SBP was already declining by age 65 y in the HAS female cohort. Compared to the population-based cohorts, SBP remained lower through midlife in the WHII occupational cohort, and the midlife acceleration in SBP occurred later (Figure 2). For example, at age 60 y, mean SBP in WHII was 17.5 mm Hg and 15.5 mm Hg lower than that of the T-07 1932/1933 cohort for men and women, respectively, and reached a velocity of 1 mm Hg per year at age 75 y in men and 65 y in women, compared with 50 y in the other cohorts.

**Table 4:** Regression coefficients (standard errors) for the fixed effects from the main unadjusted multilevel models displayed in Figure 2.

Group	Study	Intercept Age^a^	Intercept	Age	Age^2^	Age^3^
**Men**	ALSPAC	7	99.6 (0.21)	0.757 (0.211)	0.034 (0.060)	0.031 (0.005)
	T-07 1972/1973	15	113.8 (0.6)	0.897 (0.137)	−0.018 (0.006)	—
	T-07 1952/1953	35	123.2 (0.82)	0.32 (0.142)	0.019 (0.006)	—
	NSHD	36	123.9 (0.39)	0.031 (0.096)	0.038 (0.005)	—
	T-07 1932/1933	55	137.4 (1.2)	1.54 (0.220)	−0.046 (0.010)	—
	CaPS	44	135.0 (0.75)	1.27 (0.08)	−0.03 (0.002)	—
	HAS	64	158.1 (1.87)	0.40 (0.575)	−0.126 (0.036)	—
	WHII (occupational cohort)	35	125.4 (0.290)	−0.340 (0.032)	0.015 (0.001)	—
**Women**	ALSPAC	7	100.4 (0.22)	0.145 (0.218)	0.351 (0.060)	−0.008 (0.005)
	T-07 1972/1973	15	107.0 (0.56)	0.63 (0.129)	−0.005 (0.006)	—
	T-07 1952/1953	35	115.2 (0.80)	0.86 (0.134)	−0.007 (0.006)	—
	NSHD	36	118.1 (0.42)	0.37 (0.104)	0.028 (0.006)	—
	T-07 1932/1933	55	136.7 (1.01)	1.482 (0.203)	−0.348 (0.01)	—
	HAS	64	167.8 (2.6)	−0.63 (0.84)	−0.14 (0.054)	—
	WHII (occupational cohort)	35	116.7 (0.494)	0.006 (0.054)	0.017 (0.001)	—

a This is the year that age was centred to in each model.

We carried out several post hoc analyses to investigate possible reasons for the deceleration and decline seen in the older cohorts. An underestimation of the effect of HypRx was unlikely to have caused an artefact in the patterns we observed in old age (see Text S1). Excluding individuals who had ever taken HypRx in order to capture the trajectories in a healthy untreated sub-group explained a large part of the declining pattern observed at older ages (see Text S4). For example, in the T-07 1932/1933 cohort, the SBP of this non-medicated group continued to rise in a linear manner through old age, and in CaPS, the average untreated trajectory continued to rise for a longer period in later life and reached a plateau rather than exhibiting decline as in the primary analyses including treated individuals. The additional exclusion of those individuals who had suffered a myocardial infarction in this cohort further emphasised this plateau. Survivor bias (i.e., survival of those who are most healthy and least prone to common causes of premature mortality such as CVD) was unlikely to explain the general declining pattern of SBP, as the average trajectory of those who were still alive at the last wave of measurement still showed a deceleration and decline in the older population-based cohorts (see Text S4).

### BMI Adjusted Trajectories

**Figure 3:**
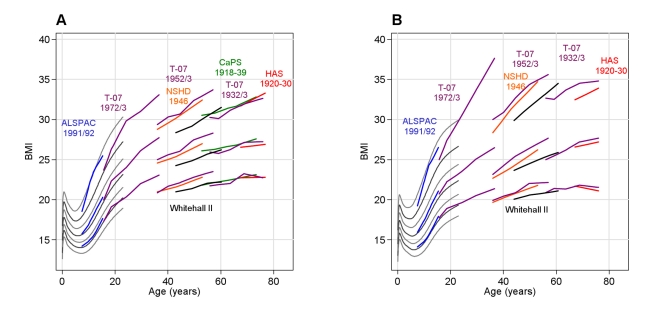
Observed BMI (kg/m^2^) in each cohort. The lines represent the median, 10^th^ and 90^th^ centile at each wave in each cohort in men (A) and women (B). The grey lines from 0 to 23 y are centiles from the UK 1990 growth reference (see reference 26) spaced approximately 2/3 of a standard deviation apart (2^nd^, 10^th^, 25^th^, 50^th^, 75^th^, 90^th^ and 98^th^ centiles).

ALSPAC children were taller than the T-07 1972/1973 cohort at baseline when compared on the UK 1990 growth scale (+0.28 *z* and +0.16 *z* in ALSPAC versus T-07 boys and girls, respectively; Table 3). The median BMI (UK 1990 *z*-score) was similar in these cohorts, but individuals in the upper centiles of ALSPAC had a larger BMI than those in the upper centiles of T-07 1972/1973 (Figure 2). At age 15 y, SBP in ALSPAC was ∼10 mm Hg higher than in T-07. Adjusting the SBP trajectory to the UK 1990 growth reference made little difference to this cohort difference (Figure 3).

**Figure 4:**
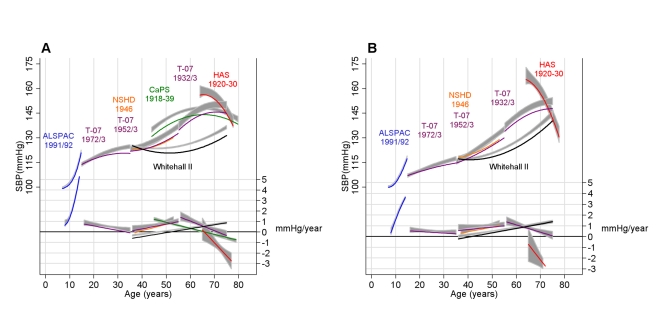
Predicted mean SBP and velocity after adjusting for BMI. Coloured and black lines are the predicted mean SBP trajectory (in millimetres of mercury) and velocity (millimetres of mercury per year) after adjusting for BMI as a time-varying covariate (see Methods and Text S2 for full details of this adjustment) in men (A) and women (B). The grey areas are the 95% CIs from unadjusted models.

BMI increased through adult life in all cohorts, with steeper rises seen in early to mid adulthood (20 to 50 y) (Figure 3). Adjusting the SBP trajectory for BMI at each age in each cohort appeared to slow some of the SBP rise seen between 30 and 40 y, but the biggest impact of the BMI adjustment was on the intercept, shifting each cohort's mean SBP trajectory downwards (Figure 4). Table 5 shows the association between BMI and SBP in each cohort. Among adult cohorts there was a suggestion that the association was largest at 50 to 60 y.

**Table 5:** Association (β) between concurrent BMI (per *z-*score increase in UK 1990 growth reference units in cohorts where data collection began in childhood or adolescence and per kilogram/metre^2^ in adult cohorts) and SBP (millimetres of mercury) in each cohort based on models including BMI as a time-updated covariate.

Sex	Study	Age	β	95% CI
**Male**	ALSPAC^a^	7 to 16	2.29	2.12, 2.46
	T-07 1972/1973^a^	15 to 37	3.63	3.01, 4.24
	T-07 1952/1953	34 to 60	0.85	0.59, 1.11
	NSHD	40	0.65	0.48, 0.82
		50	1.07	0.90, 1.25
	WHII^b^	40	1.00	0.89, 1.12
		50	1.18	1.09, 1.27
		60	1.18	1.07, 1.29
		70	0.93	0.67, 1.19
	T-07 1932/1933	55 to 77	1.07	0.72, 1.43
	HAS	63 to 81	0.56	0.04, 1.08
	CaPS^b^	50	1.38	1.17, 1.59
		70	0.92	0.72, 1.10
**Female**	ALSPAC^a^	7 to 16	2.53	2.36, 2.70
	T-07 1972/1973^a^	15 to 37	2.73	2.18, 3.28
	T-07 1952/1953	34 to 60	1.01	0.83, 1.20
	NSHD	40	0.47	0.33, 0.61
		50	0.76	0.63, 0.89
	WHII^b^	40	0.54	0.40, 0.69
		50	0.82	0.72, 0.92
		60	0.86	0.74, 0.99
		70	0.61	0.33, 0.90
	T-07 1932/1933^b^	60	1.08	0.81, 1.34
		70	0.56	0.27, 0.84
	HAS	63 to 81	0.49	−0.09, 1.08

a Beta is per *z*-score increase on the UK 1990 growth reference scale (28).

b Results are presented at several ages in cohorts where there was evidence that the association between concurrent BMI and SBP differed across age.

### Sex Differences

**Figure 5:**
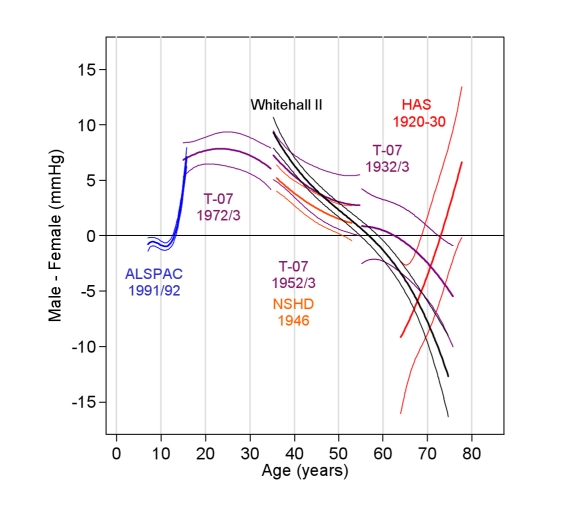
Mean sex difference in SBP (men minus women) (in millimetres of mercury) and 95% CIs. Estimated from multilevel models adjusting for current BMI (all cohorts) and with additional adjustment for baseline height in the child cohorts (ALSPAC and T-07 1972/1973). Positive values indicate a higher SBP in males.

Figure 5 shows the sex differences in SBP in each cohort. The pattern of these differences was unaffected by adjustment for BMI (unadjusted not shown). From age 7 to 12 y, ALSPAC girls had a slightly higher SBP; boys caught up by age 13 and then overtook girls such that by age 15 y the mean SBP in boys was 5.0 mm Hg higher (95% CI: 4.5, 5.6). Similar sex differences were seen at age 15 y in the T-07 1972/1973 cohort. The maximum sex difference occurred at age 26 y (+8.2 mm Hg in men, 95% CI: 6.7, 9.8) in the T-07 1972/1973 cohort. Women experienced steeper early adulthood and midlife rises, and by the sixth decade there was no evidence of a sex difference in SBP in the T-07 1932/1933 cohort. However, in the HAS cohort, women had a higher SBP from 64 to 72 y, and men had a higher SBP from age 72 y. The pattern of sex difference in WHII followed the trends in the population-based cohorts.

## Discussion

### Main Findings

Our analysis describing the unadjusted pattern of SBP over life in population-based studies showed four chronological phases: (1) a rapid increase in SBP coinciding with peak adolescent growth, (2) more gentle increases in early adulthood, (3) a midlife acceleration beginning in the fourth decade, and (4) a period of deceleration in late adulthood, where increases in SBP slowed. These phases were not explained by increases in BMI through adulthood, but the deceleration in old age was less evident when restricting the analyses to individuals who had never taken antihypertensive therapy. Compared to the population-based cohorts, the occupational cohort (WHII) had a lower mean SBP, a shallower annual increase in midlife, and a later midlife acceleration. Men and women had different life course trajectories but a similar mean SBP by the seventh decade—females had lower increases during adolescence, but steeper rises from early to mid adulthood. Given population differences in SBP [29], our results may not be generalisable to populations with a different distribution of BP-related exposures.

### Comparison with Other Studies

The mean level of SBP and annual increases found in childhood and adolescence were within the range seen in population-based cross-sectional data from the UK [30] and in longitudinal data from the US [13]. These increases are typically viewed as a concomitant of growth in size and stature. Cross-sectional [10],[31] and longitudinal data [14],[16],[32] in western populations also substantiate the gentle increases in SBP from early adulthood and the midlife acceleration in the fourth decade.

In WHII, SBP was similar to that of the population-based cohorts at age 35 y, but the midlife increases occurred later, resulting in a lower mean SBP through to late adulthood. These differences are supported by the lower mortality rate in WHII compared to the general UK population [33]. WHII can be seen as a population sub-group nested within the UK population at large. Similar patterns of BP differences can also be seen in within-population comparisons from the US [34].

These unadjusted mean trajectories reflect both any underlying effects of ageing together with the distribution of lifetime BP influencing exposures in each cohort. To test how a strong determinant of BP might affect the shape of the trajectory, we adjusted for concurrent BMI at each age. The associations between BMI and SBP were similar in these observational studies to those reported in randomised controlled trials and Mendelian randomisation studies [8],[17]. We found that although mean SBP was lower after adjustment for BMI, BMI did not greatly influence the magnitude of age-related increases in SBP. One possible exception was the period around 30 to 40 y, where the rate slowed after adjustment—this was also the period of most rapid weight gain in our cohorts, which might explain this finding.

Similar to our study, other population-based longitudinal studies in western societies have reported a deceleration and a decline of SBP in old age (>65 y) [16],[35]–[37]. Some cross-sectional studies did not show a decline [9],[38], although this may be partly obscured by secular decreases in BP [10],[11]. Our sensitivity and post hoc analyses (see Text S4) suggested that incorrect adjustment for HypRx in old age or survivor bias were unlikely to explain this general pattern in old age. However, some of the deceleration and decline was no longer evident after excluding individuals who had taken HypRx. This finding is in line with previous studies that have made similar exclusions in order to capture BP trajectories in healthy individuals [16],[39], and is supported by studies that have shown an association between SBP decline and higher mortality among the elderly [35]. A decline in SBP in old age has been linked to deteriorating health [35],[40], and a low BP to impaired cardiac output [41], suggesting decreasing BP is a feature of ageing in western populations. The deceleration and decline was not yet apparent in the WHII cohort, which may be a reflection of the better health and lower mortality of this cohort [33].

The steep declines in SBP in the HAS cohort were larger than those in other studies [16],[35]–[37], particularly in women, and were less reduced in the healthy sub-sample. These results should be interpreted with caution because of the small sample size and reliance on only one follow-up measure of BP. It is also possible that the trajectory in HAS is affected by systematic measurement differences between the two waves [42], and if so, errors in the estimation of the trajectory are likely to be exaggerated when based on only two observation points.

### Gender Differences

Similar to our study, other longitudinal studies have shown boys to have greater SBP rises during adolescence [13] and women to have steeper rises from midlife [14],[16],[37]. The latter pattern has also been shown in aggregated cross-sectional data from almost all World Health Organization global sub-regions [38]. One exception to this pattern is in unacculturated societies such as the Yi in China [43], where gender differences were not observed. Heightened sodium sensitivity due to hormonal changes accompanying menopause is one proposed pathway to explain the gender dimorphism of BP around midlife [44]. The lack of gender differences in isolated populations where salt intake is low supports such a mechanism [43].

### Interpretation and Implications

Given the general similarity in measurement methods, birth years, and period of data collection, it seems reasonable to attribute some of the more favourable BP trends in WHII compared to the other cohorts to socially patterned and modifiable BP-related exposures such as lifestyle and diet [45] that act across the life course. Selection on the basis of being fit enough to work is also likely to mean that this cohort was healthier at baseline than the general population. And it is possible that either because of their higher SEP or as a result of work-based initiatives, participants in this cohort may be more likely to be treated earlier with antihypertensive therapy. This is suggested by the fact that the prevalence of HypRx use in this cohort in midlife was similar to that of other cohorts crossing the same age period, but the average BP in WHII was lower. However, some of the differences in WHII could be explained by variation in BP measurement, although given the differences in the shape of the trajectory, it seems unlikely that this would explain all of the disparity.

Our study was not designed to answer how much of the increase in SBP with age is a natural physiological feature of ageing. There are a number of cross-sectional studies in isolated communities that show virtually no age-related increase in BP [43]. These communities typically have a predominantly vegetarian diet with very low salt content, physically arduous lifestyles, and very low or non-existent levels of obesity. One study also showed that individuals who had undergone a rural to urban migration from one of these isolated communities quickly went on to adapt the BP profiles of their adopted communities [43]. The stronger effect of age-related BMI increases on the intercept rather than slope of the SBP trajectory seen in our analysis might also reflect the importance of factors other than BMI, as shown by the opposing secular trends of falling BP but rising obesity levels seen in recent decades [10]–[12]. The trajectories in each cohort can be seen as a reflection of the dynamic aggregate of lifetime BP-related exposures. In this sense, one might expect the population-based trajectories to take a form more similar to that of WHII if the general population had similar lifetime exposures and ways of living.

The midlife acceleration in SBP is interesting because of the transition from a period of flatter increases in early adulthood. Several studies suggest that individuals with a high SBP in midlife are at risk of more rapid arterial ageing, characterised by a stiffening of the large arteries [14],[46]. In contrast, the smaller changes seen in early adulthood may reflect more capacity for vascular repair or adaptation at this age [47],[48]. The delayed acceleration in WHII and widening distribution of SBP from 40 to 60 y might indicate that this is a point of transition when some individuals experience an earlier rise. Understanding the variation in midlife trajectories and factors driving this acceleration may be important for understanding the development and prevention of CVD risk. This is of particular importance given that a prolonged shift of just a few millimetres of mercury in SBP could remove a substantial burden of CVD at a population level [1].

### Strengths and Limitations

A key strength of our investigation is the use of individual-level longitudinal data and formal statistical modelling to describe SBP trajectories. The measurement protocols were relatively well standardised within each cohort, and we showed through a series of sensitivity analyses that our findings with regards to the slope of SBP were unlikely to be qualitatively affected by within- and between-cohort differences in the methods of BP measurement. However, comparisons of the mean SBP at overlapping ages between cohorts may still be subject to bias because of device differences and other unaccounted for methodological variation [42], hence the difficulty of attributing cohort or period effects. Thus, while we saw large differences in mean SBP at age 15 y between the ALSPAC and T-07 cohorts that were not explained by BMI or adolescent growth, we cannot rule out the contribution of device effects [42] because we could find no appropriate device conversion equations for younger populations.

Despite the measurement issues, the use of multiple cohorts with longitudinal data to make inference on the change in BP over life seems viable when the methodology has been well standardised across waves. An extension to this approach is to join the trajectories from each cohort under certain assumptions and study the effect of lifetime exposures on the lifetime trajectory. This will require methodological development, but the use of this approach with commonly collected measures of biological function such as BP could provide a better and more dynamic understanding of when and how in the life course health is compromised.

### Conclusion

We have described several lifetime phases in the age-related progression of SBP and have shown that the typical increases in BMI that accompany ageing are more strongly related to the mean BP than to the age-related changes in BP that we see in our population. We have also shown sex differences in BP change that are consistent with the hypothesis of a menopause-related effect on salt sensitivity. Lastly, our results provide some evidence that an occupational cohort with generally higher SEP than the general UK adult populations studied here has a slower midlife increase in BP and hence lower average levels in their 40s , 50 s, and 60 s. Whilst our study is unable to identify the key determinants of age-related increases in BP, it does suggest that these are modifiable, but perhaps not by exposures that largely influence BP through the increases in BMI that tend to accompany ageing. Further research should try to understand which factors affect this trajectory and when in the life course such factors exhibit most influence.

## Supporting Information

Text S1 Sensitivity analysis of device and treatment effects.
(0.04 MB PDF)

Text S2 More details on the multilevel models.
(0.06 MB PDF)

Text S3 Missing data analyses.
(0.02 MB PDF)

Text S4 Post hoc analysis examining reasons for the patterns of SBP in late life.
(0.04 MB PDF)

## Abbreviations

ALSPAC: Avon Longitudinal Study of Parents and Children
AO: automated oscillometric
BMI: body mass index
BP: blood pressure
CaPS: Caerphilly Prospective Study
CVD: cardiovascular disease
HAS: Hertfordshire Ageing Study
HypRx: antihypertensive drug(s)
MRZ: manual random zero sphygmomanometer
NSHD: National Survey of Health and Development
SBP: systolic blood pressure
SEP: socioeconomic position
T-07: West of Scotland Twenty-07 study
WHII: Whitehall II study
