# Supplementary material for: Life Course Trajectories of Systolic Blood Pressure Using Longitudinal Data from Eight UK Cohorts
Source: PLoS Med. 2011 Jun 14;8(6):e1000440. doi: 10.1371/journal.pmed.1000440 (PMC3114857; doi:10.1371/journal.pmed.1000440)
Supplement: Text S1 — Sensitivity analysis of device and treatment effects. (0.04 MB PDF) [file pmed.1000440.s001.pdf]

Text S1. Sensitivity analysis of machine and treatment effects

1.1 Ignoring the machine switch in later waves:

Figure S2 shows the effect of not converting the SBP values for the later waves in the T-07, NSHD and CaPS cohorts where the machine was switched to an AO device. Failing to correct for the machine switch distorted the life course SBP trajectory in each cohort by making the trajectory steeper at ages when the machine was switched, particularly in men (figure S1). It made little difference in the T-07 (1972/3) female cohort.

1.2 Influence of type of blood pressure measurement device:

The sensitivity of our results to the type of blood pressure (BP) device used was assessed by refitting models in the T-07 cohorts using the unconverted automated oscillometric (AO) readings from the final 3 waves and comparing them to the main analysis that used the converted measures. Since the AO measures were taken at the 2nd reading in wave 3 (Table 1 – main paper) which are on average lower than the 1st, we estimated an equivalent 1st reading measure using a linear regression equation developed from the wave 4 data by regressing the 1st reading on the 2nd. For this analysis, we expected a vertical shift in the AO intercept compared to the manual random zero sphygmomanometer (MRZ). However, we were more concerned with how the change of instrument affected the slope of SBP since this was the main focus of the paper.

Figure S2 shows that SBP from the AO device did overestimate the mean SBP compared to the MRZ device. However, with the exception of the early adult period

(24-35 years) in females, the slopes of the trajectory were qualitatively similar to those estimated using the MRZ and converted values.

1.3 Differences between the 1st and 2nd BP readings:

Figure S3 shows the estimated SBP trajectories when using the 1st and 2nd BP readings (CaPS is not shown since it only had one reading for the majority of waves - see Table 2). While the 2nd BP reading had a lower mean compared to the 1st, the choice of replicate made little difference to the general age-related pattern of SBP. The annual increase was slightly less at older ages when using the 2nd replicate. The use of the 2nd reading shows the period of deceleration in late adulthood more strongly than the 1st reading.

1.4 Choice of treatment effect:

In the main paper we used a treatment effect of 10mmHg. We did a sensitivity analysis allowing for a 15mmHg and 20mmHg treatment effect to check the robustness of our findings to this choice. Figure S4 shows that the choice of constant for treatment effect did not qualitatively alter our findings on the general life course pattern of SBP, and in particular the deceleration and eventual decline at older ages remained.

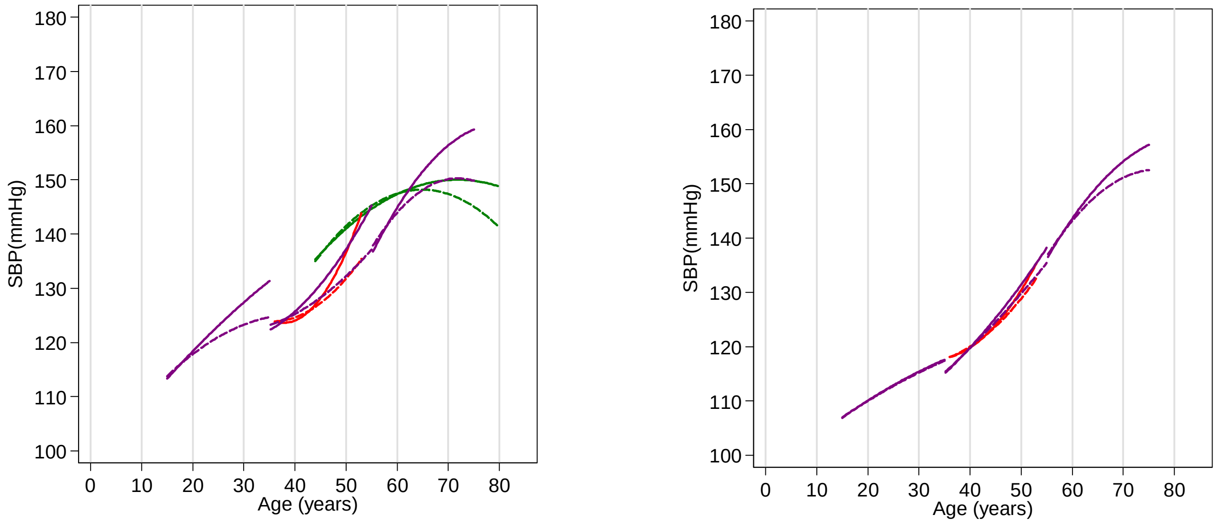

Figure S1. Effect of not converting the SBP from waves when the machine was switched. Predicted mean trajectories of SBP without converting the later waves from an AOD to RZ device (solid line). To allow a comparison, the predicted mean from the converted measures (from figure 2) is re-plotted (dashed line).

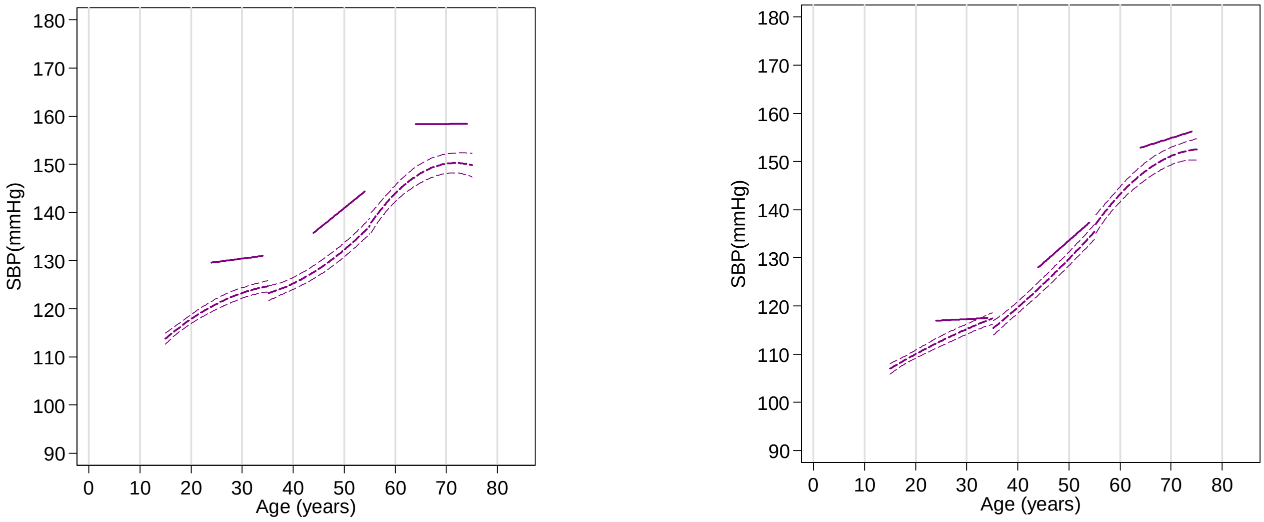

Figure S2. MRZ versus AOD. Predicted mean SBP in the T-07 cohorts using the last 3 waves where an AOD was used in men (left) and women (right). To allow a comparison, the SBP trajectory and 95% CI from figure 2 (using the converted measures at waves 3-5) are re-plotted (dashed lines).

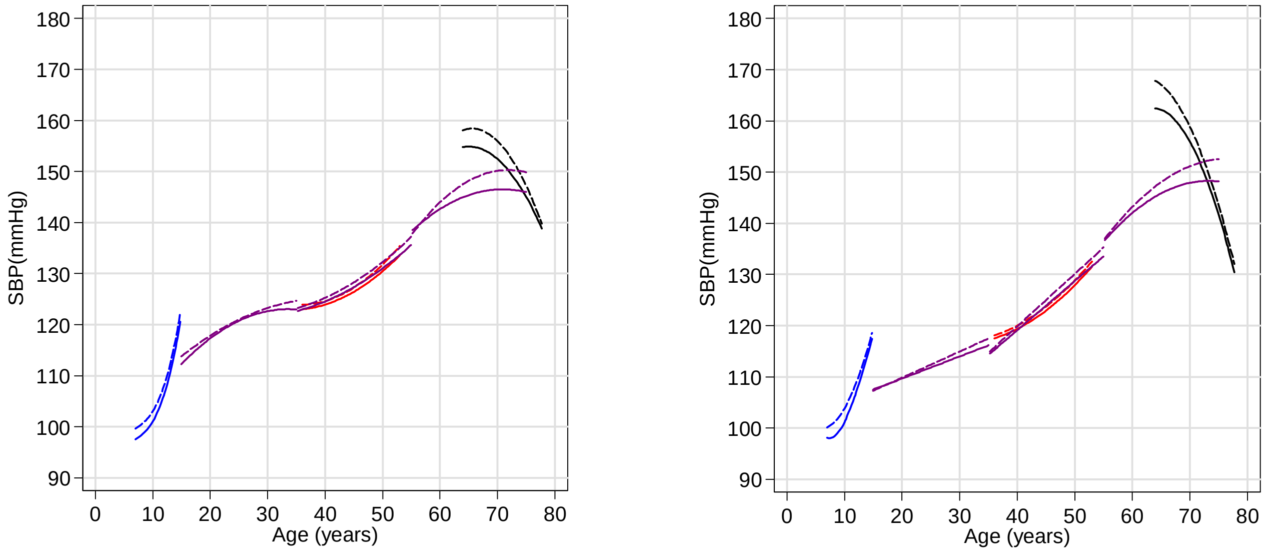

Figure S3 First v second BP replicate.
Predicted mean SBP in each cohort when using the 2nd replicate BP measurement (solid line). To allow a comparison, the predicted mean from the converted measures (from figure 2) is re-plotted (dashed line).

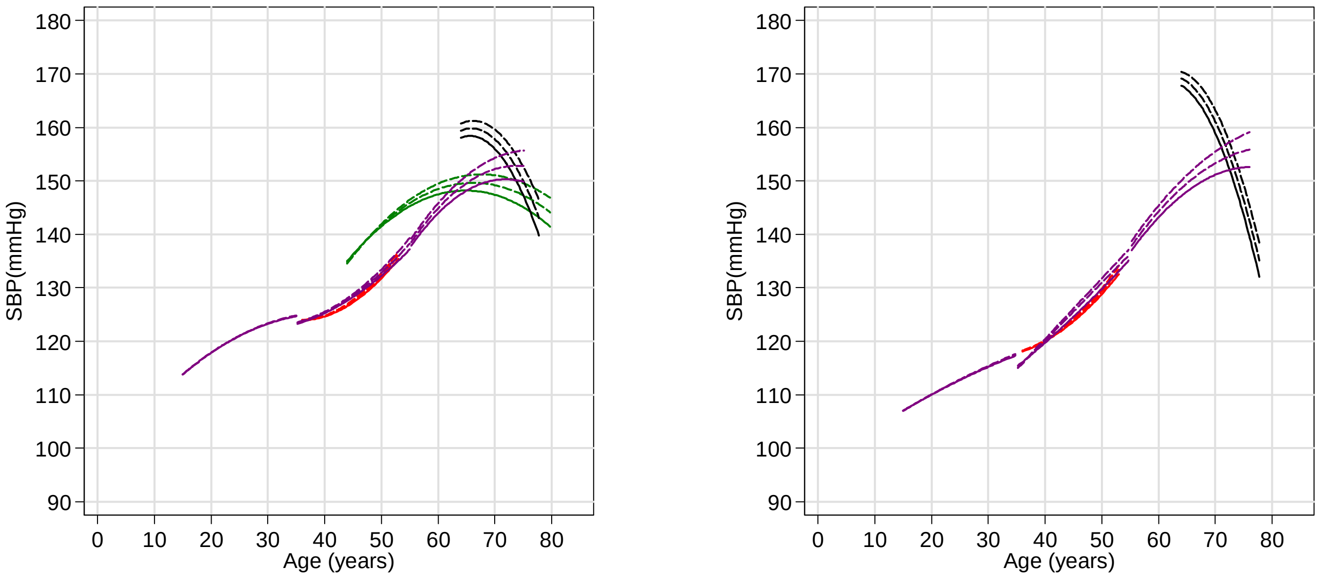

Figure S4. Choice of treatment effect.
The dashed lines are from unconditional models assuming a 15mmHg and 20mmHg treatment effect and the solid lines a 10mmHg reduction (as presented in the main paper).
