# Supplementary material for: Life Course Trajectories of Systolic Blood Pressure Using Longitudinal Data from Eight UK Cohorts
Source: PLoS Med. 2011 Jun 14;8(6):e1000440. doi: 10.1371/journal.pmed.1000440 (PMC3114857; doi:10.1371/journal.pmed.1000440)
Supplement: Text S2 — More details on the multilevel models. (0.06 MB PDF) [file pmed.1000440.s002.doc]

**Text S2. More details on the multilevel models**

The following model was considered:

SBP_ij_= β_0_ + μ_0i_ + (β_1_ + μ_1i_) age_ij_ + (β_2_ + μ_2i_) age^2^_ij_ + (β_3_ + μ_3i_) age^3^_ij_ + ε_ij_ (1)

Where SBP_ij_ is the systolic blood pressure for individual *i* at observation time *j*. β_0_ and β_1_ are the fixed intercept and slope, μ_0i_ to μ_3i_ are the random intercept and slope coefficients for each individual *i*, and ε_ij_ is the residual error term for individual *i*, at time *j*. Covariance between the random coefficients was allowed . Age was centred at the baseline age in each cohort (see Table 4). The random intercepts and slopes allow for the changing variance with age.

A cubic polynomial was considered sufficiently flexible to describe the physiological progression of SBP within each cohort. The final and most parsimonious model was chosen by removing the non-significant higher order terms. A decision was made a priori not to select models that had a period of decreasing SBP followed by a period of increasing SBP at the edges of the data since this pattern is biologically implausible and is likely to reflect over fitting to observations at the youngest and oldest ages and/or over fitting to systematic measurement differences between waves. Random coefficients for the powers in the polynomial were also included and evaluated in terms of improvement in model fit using likelihood ratio tests.

BMI adjusted models:

For ALSPAC and T-07 cohorts whose first data collection wave began in childhood, the following model was fitted:

SBP_ij_= β_0_ + μ_0i_ + (β_1_ + μ_1i_) age_ij_ + (β_2_ + μ_2i_) age^2^_ij_ + (β_3_ + μ_3i_) age^3^_ij_

+ γ_1_ zBMI_ij_ + γ_2_ (age_ij_ zBMI_ij_) + γ_3_ (age^2^_ij_ zBMI_ij_)

+ δ_1_ zbHT_i_ + δ_2_ (zbHT_i_ age_ij_) + δ_3_ (zbHT_i_ age^2^_ij_) + δ_4_ (zbHT_i_ age^3^_ij_)

+ ε_ij_ (2)

Here, zBMI_ij_ is the BMI z-score externally referenced to the UK 1990 sex and age-specific growth charts (1) for subject *i* at observation time *j*. γ_1_ thus represents the cross sectional association between BMI and SBP and γ_2_ and γ_3_ allow the association between current BMI and SBP to vary by chronological age. zbHT_i_ represents the height z-score at baseline referenced to the UK 1990 sex and age-specific growth charts (1) for subject *i*. δ_1_, δ_2_ and δ_3_ thus allow baseline height to affect the SBP intercept and slope. Non significant γ’s and δ’s were removed from the final model. This formulation attempts to adjust SBP in the ALSPAC and T-07 (1972/3) cohorts to the same BMI and height/developmental growth trajectory, that is to the UK 1990 growth reference (1). Importantly we don’t attempt to remove the influence of growth per se, as this would be over adjusting, but rather to adjust growth to a common external standard in each cohort.

For the older cohorts (T-07 1952/3, T-07 1932/3, NSHD, CAPS, HAS and WHII), the following model was fitted:

SBP_ij_= β_0_ + μ_0i_ + (β_1_ + μ_1i_) age_ij_ + (β_2_ + μ_2i_) age^2^_ij_ + (β_3_ + μ_3i_) age^3^_ij_

+ γ_1_ cBMI_ij_ + γ_2_ (age_ij_ cBMI_ij_) + γ_3_ (age^2^_ij_ cBMI_ij_)

+ ε_ij_ (3)

Here cBMI_ij_ is BMI centred at 23kg/m^2^ for subject *i* at observation time *j*. This model attempts to control for the increase in BMI that is commonly seen with ageing so that β_0_ to β_3_ now describe the age-related trajectory of SBP in a population with a constant BMI of 23 kg/m^2^ through adult life.

The reason that BMI was modelled using a time-varying covariate was because studies have shown that BMI, or factors that affect blood pressure that are picked up by BMI, has relatively acute effects on blood pressure. For example, see randomised controlled trials of the effects of weight loss on blood pressure eg, (2); and life course studies that have examined the influence of BMI on blood pressure at different ages and shown that current BMI captures most of the variability in blood pressure eg, (3).

Reference List

(1) Cole TJ, Freeman JV, Preece MA. British 1990 growth reference centiles for weight, height, body mass index and head circumference fitted by maximum penalized likelihood. Stat Med 1998; 17(4):407-429.

(2) Neter JE, Stam BE, Kok FJ, Grobbee DE, Geleijnse JM. Influence of Weight Reduction on Blood Pressure: A Meta-Analysis of Randomized Controlled Trials. Hypertension 2003; 42(5):878-884.

(3) Wills AK, Hardy RJ, Black S, Kuh DJ. Trajectories of overweight and body mass index in adulthood and blood pressure at age 53: the 1946 British birth cohort study. J Hypertens 2010; 28(4):679-686.
