# Supplementary material for: Life Course Trajectories of Systolic Blood Pressure Using Longitudinal Data from Eight UK Cohorts
Source: PLoS Med. 2011 Jun 14;8(6):e1000440. doi: 10.1371/journal.pmed.1000440 (PMC3114857; doi:10.1371/journal.pmed.1000440)
Supplement: Text S3 — Missing data analyses. (0.02 MB PDF) [file pmed.1000440.s003.pdf]

Text S3. Missing data analyses

Most of the missing data patterns were monotonic in the sense that once an individual dropped out of the study they were less likely to return. We thus compared the characteristics of this group with those who remained in the study or had returned by the last recorded wave. Table S1 shows the mean differences between groups in terms of the variables described in the main paper. Generally, individuals who dropped out and didn't return were more likely to have a higher SBP and be on medication for high blood pressure at baseline. BMI also tended to be greater in those not followed up, although with the exception of the NSHD, these differences were small (<0.6kg.m^2). There were some differences in baseline height, for example, those not followed up in the CaPS cohort were on average 1.7cm shorter. Other height differences were small. In all of the cohorts except the HAS and T-07 (1952/3) females, participants from lower socioeconomic groups were more likely to drop out.

The multilevel models used in our study give unbiased estimates under a missing at random (MAR) assumption (1) if variables that predict the missingness are conditioned on. To explore possible bias under MAR, the models were refitted allowing baseline BMI, height and social class to affect the intercepts and slopes. Some of these covariates are associated with attrition in each study (Table S1). Figure S5 shows the estimated mean SBP trajectories from these models and the mean SBP from a set of unconditional models on the same sample. There was little difference in the intercepts and slopes between the two sets of models in each cohort. This provides some support that our main models for SBP shown in figure 1 are unlikely to be biased if the data are missing at random. However, there may be other unmeasured

variables that are important predictors of the missingness, and this does not rule out potential bias if the data do not meet the assumptions of missing at random.

Table S1. Missing data: Mean difference between individuals who dropped out of the study and didn't return, and individuals who stayed in the study. Positive values indicate higher values in those who went missing. P-values are from a t-test for continuous variables, and a chi-squared test for proportions.

	**SBP (mmHg)**		**Medication (%)**		**Height (cm)**		**BMI (kg.m^2)**		**Adult SEP (%)†**		**Childhood SEP (%)†**	
	**Diff**	**p**	**Diff**	**p**	**Diff**	**p**	**Diff**	**p**	**Diff**	**P**	**Diff**	**p**
**CaPS**												
Men	+4.3	<0.001	+4.3	<0.001	-1.7	<0.001	+0.09	0.6	+15.2	<0.001	+4.6	0.004
**HAS**												
Men	+2.3	0.27	+3.7	0.41	-0.9	0.17	+0.03	0.9	+13.4	0.0047	+5.2	0.15
Women	+7.5	0.008	+10	0.052	-0.3	0.66	-0.56	0.28	-1.4	0.8	-1.1	0.79
**NSHD**												
Men	+2.2	0.02	+6.8	0.29	+0.2	0.67	+0.39	0.048	+7.9	0.0073	+5.1	0.09
Women	+1.8	0.066	+2.8	0.74	-1.6	0.034	+1.0	<0.001	+7.1	0.0223	+5.1	0.11
**T-07 (1932/3)**												
Men	-0.4	0.9	+5.5	0.105	-0.9	0.16	+0.12	0.7	+12.8	0.007	-	-
Women	+0.4	0.8	+9.3	0.007	-1.2	0.03	+0.55	0.22	+10.7	0.017	-	-
**T-07 (1952/3)**												
Men	+1.7	0.32	+4.6	0.027	-0.8	0.34	+0.37	0.34	+13.6	0.0177	-	-
Women	+1.9	0.21	+2.3	0.26	-0.3	0.61	+0.19	0.7	+7.8	0.12	-	-
**T-07 (1972/3)**												
Men	+1.2	0.35	(a)	(a)	-0.6	0.4	-0.27	0.35	NA	NA	+10.1	0.039
Women	-1.2	0.33	+6.2	0.18	0.0	0.96	-0.03	0.9	NA	NA	+13.3	0.007
**ALSPAC**												
Boys	+0.92	<0.001	NA	NA	-0.1	0.47	+0.13	0.055	NA	NA	+11.4	<0.001
Girls	+0.2	0.6	NA	NA	+0.1	0.6	+0.22	0.004	NA	NA	+6.4	<0.001

† SEP: the proportion of people in manual social classes.
NA: not applicable.
(a) zero cell count.

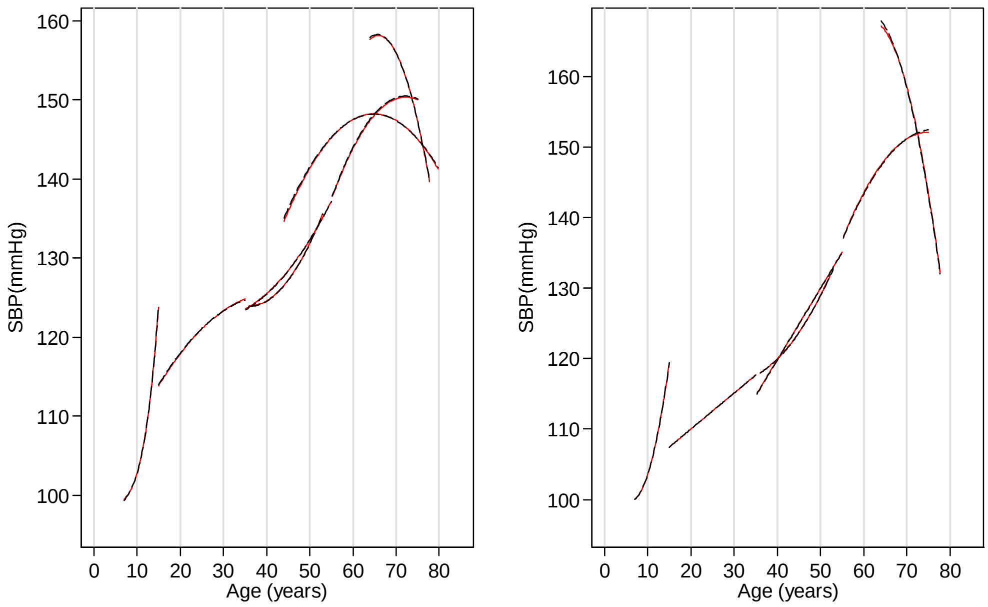

Figure S5. Investigation of bias under MAR. Predicted mean trajectories of SBP with adjustment of baseline BMI and height, and current social class (red solid line) and in unconditional models (black dashed line). Both sets of models were estimated using the same sample.

<u>Reference</u>

(1) Molenberghs G, Kenward MF. Missing data in clinical studies. 1st ed. Chichester: John Wiley and Sons Ltd, 2007.
