# Supplementary material for: Life Course Trajectories of Systolic Blood Pressure Using Longitudinal Data from Eight UK Cohorts
Source: PLoS Med. 2011 Jun 14;8(6):e1000440. doi: 10.1371/journal.pmed.1000440 (PMC3114857; doi:10.1371/journal.pmed.1000440)
Supplement: Text S4 — Post hoc analysis examining reasons for the patterns of SBP in late life. (0.04 MB PDF) [file pmed.1000440.s004.pdf]

Text S4. Post hoc analysis examining reasons for the patterns of SBP in late life

<u>S4.1 Trajectories in a healthy sample</u>

Some studies have shown an association between a decline in SBP and poor health and/or disease (1;2). To assess whether the deceleration and decline may be associated with poorer health and treatment we excluded all those individuals who had ever been prescribed antihypertensive medication (HypRx), ie., a less healthy subset, and then refitted the models. If part of the deceleration and decline in old age is linked with health, we expected to see some of this pattern removed in the healthier subsample. In CaPS we also had information on myocardial infarctions (MI), so refitted another model with the additional exclusion of anyone who had experienced an MI.

Figure S6 shows the results from these models. In all of the older cohorts the decline was reduced. In the T-07 cohorts, the deceleration was no longer evident and linear increases through to age 75 years were seen. In the CaPS cohort the decline that was present when using the whole population sample had almost completely disappeared after excluding those who had taken HypRx, further exclusion of those who had an MI emphasised this finding a little more. However, the deceleration or slowing of age-related rises in SBP was still present in CaPS. In HAS there was still a decline but the decline was smaller in the restricted healthy subset.

<u>S4.2 Survival of the fittest:</u>

Survivor bias (i.e. survival of those who are most healthy and least prone to common causes of premature mortality such as cardiovascular disease) might result in a decline

at older ages. The models used in this study give unbiased estimates if the missing data are dependent only on the observed SBP and not on the unobserved. This assumption is reasonable for the younger cohorts, but for the older cohorts it is likely that individuals with a higher future SBP are less likely to be observed later on given the strong association of high BP with mortality (3;4). In this scenario, the trajectory at older ages will reflect survivorship rather than the ageing process. We investigated the average SBP trajectory in individuals who had survived the longest by restricting the analysis to those who were still in the study and hence still alive at 70 years in the T-07 (1932/3), CaPS and HAS cohorts. Since 70 years was a somewhat arbitrary cut-off based on the ages where we had data, we repeated this analysis in the sample who had data and had hence survived beyond 75 years.

The sample size was small in these models for the T-07 and HAS cohorts -there were 230 men and 196 women in T-07, and 160 men and 224 women in the HAS cohort who were observed (i.e, survived) beyond 70 years; and 229 men and 184 women in T-07, and 84 men and 114 women in the HAS cohort who were observed beyond 75 years. Figure S7 shows that even in this select group of survivors there was still a pattern of deceleration and decline with old age.

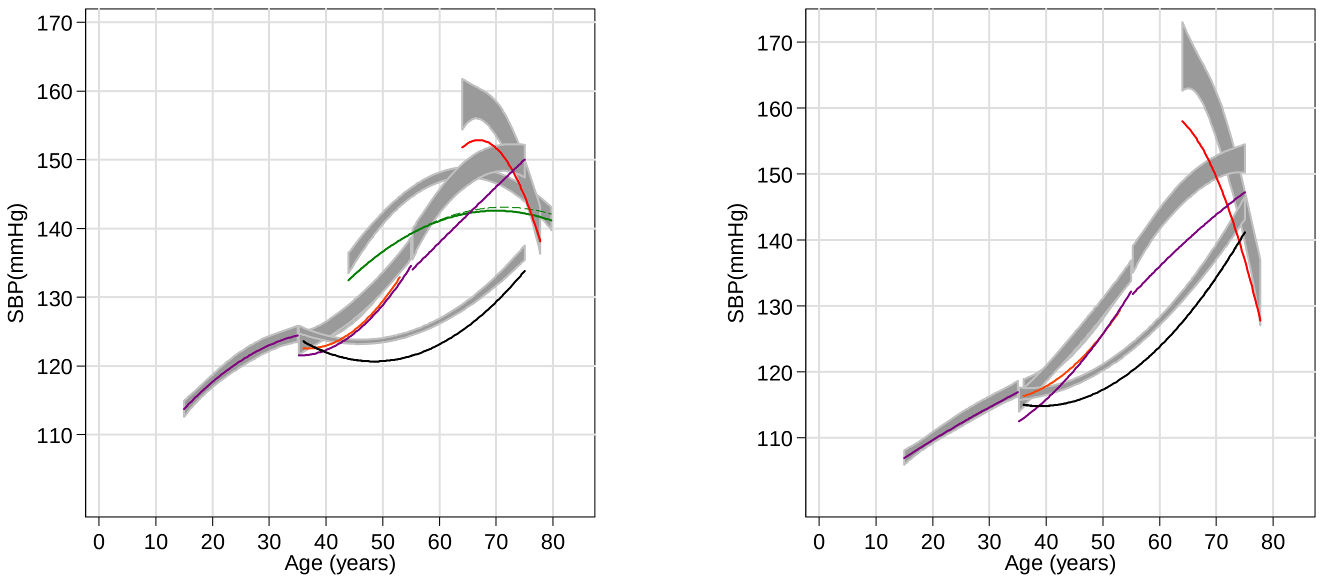

Figure S6. Predicted mean SBP after excluding individuals who had ever been treated for high blood pressure (solid line) and with additional exclusion of individuals who had suffered a myocardial infarction (dashed line – CAPS only) in men (left) and women (right). To allow a comparison, the predicted means from the main models with no censoring are also plotted (grey area) (as shown in figure 2 A&B of main paper).

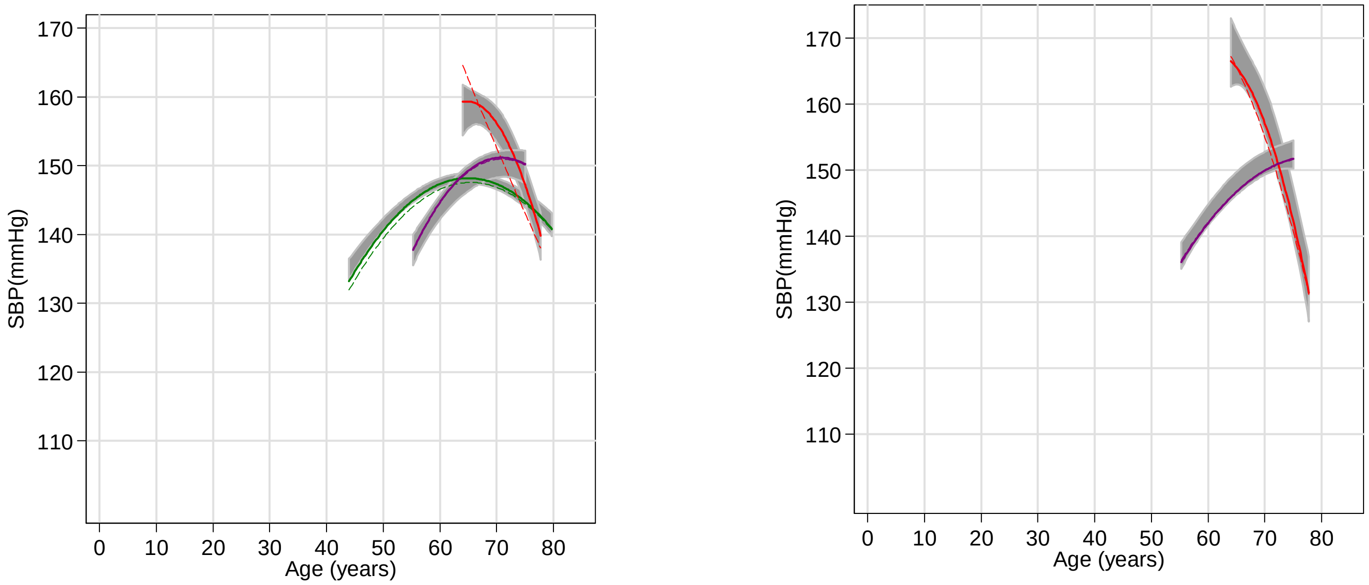

Figure S7. Survival of the fittest: Predicted mean SBP after excluding individuals who were not observed beyond 70 years (solid lines) and 75 years (broken lines) in the T-07 (1932/3), CaPS and HAS cohorts) in men (left) and women (right).

To allow a comparison, the predicted means from the main models with no censoring are also plotted (grey area) (as shown in figure 2 A&B of main paper).

Reference List

(1) Hakala SM, Tilvis RS. Determinants and significance of declining blood pressure in old age. A prospective birth cohort study. Eur Heart J 1998; 19(12):1872-1878.

(2) Starr JM, Inch S, Cross S, MacLennan WJ, Deary IJ. Blood pressure and ageing: longitudinal cohort study. BMJ 1998; 317(7157):513-514.

(3) Franklin SS, Gustin W, IV, Wong ND, Larson MG, Weber MA, Kannel WB et al. Hemodynamic Patterns of Age-Related Changes in Blood Pressure : The Framingham Heart Study. Circulation 1997; 96(1):308-315.

(4) Lewington S, Clarke R, Qizilbash N, Peto R, Collins R. Age-specific relevance of usual blood pressure to vascular mortality: a meta-analysis of individual data for one million adults in 61 prospective studies. Lancet 2002; 360(9349):1903-1913.
